# Radiotracer-Free Axillary Staging with ICG and Methylene Blue After Neoadjuvant Chemotherapy: Does Failure to Retrieve the Clipped Node Matter?

**DOI:** 10.3390/cancers18162550

**Published:** 2026-08-08

**Authors:** Merve Tokocin, Sevda Yener, Selçuk Cin, Nigar Erkoç, Eda Cingoz, Nihan Nizam Nizam, Burçin Çakan Demirel, Şahin Bedir, Turan Pehlivan, Atilla Çelik

**Affiliations:** 1 Department of General Surgery, University of Health Sciences Istanbul Bagcilar Training and Research Hospital, 34200 Istanbul, Türkiyedratillacelik@yahoo.com (A.Ç.); 2 Department of Radiation Oncology, University of Health Sciences Istanbul Bagcilar Training and Research Hospital, 34200 Istanbul, Türkiye; 3 Department of Pathology, Cerrahpasa Faculty of Medicine, Istanbul University-Cerrahpasa, 34320 Istanbul, Türkiye; selcuk.cin@iuc.edu.tr; 4 Department of Radiology, University of Health Sciences Istanbul Bagcilar Training and Research Hospital, 34200 Istanbul, Türkiye; 5 Department of Oncology, University of Health Sciences Istanbul Bagcilar Training and Research Hospital, 34200 Istanbul, Türkiye; 6 Department of Oncology, Medipol University, Medipol Mega Hospital, 34214 Istanbul, Türkiye; 7 Department of Oncology, İstinye University, Gaziosmanpaşa Medicalpark Hospital, 34250 Istanbul, Türkiye

**Keywords:** breast cancer, neoadjuvant chemotherapy, targeted axillary dissection, sentinel lymph node biopsy, clipped node

## Abstract

***Background***: Radiotracer- and magnetic seed-based targeted axillary staging techniques are increasingly used after neoadjuvant chemotherapy (NAC) in patients with initially node-positive breast cancer who convert to clinical node-negative (ycN0) status. However, these technologies remain unavailable in many centres worldwide. We evaluated the effectiveness of radiotracer-free axillary staging using dual-tracer sentinel lymph node biopsy (SLNB) with indocyanine green (ICG) and methylene blue, with particular focus on the clinical implications of clipped node non-retrieval. ***Methods***: This retrospective single-centre cohort study included 49 patients with biopsy-proven cN1 breast cancer who achieved ycN0 status following NAC and underwent dual-tracer SLNB using ICG and methylene blue between 2021 and 2024. Clipped node retrieval rates, tracer-specific detection patterns, recurrence outcomes, and disease-free survival (DFS) were analysed. Clinical outcomes, including recurrence and disease-free survival, were compared between patients with successful and unsuccessful clipped node retrieval. ***Results***: Clipped node retrieval was achieved in 41 of 49 patients (83.7%). ICG outperformed methylene blue for clipped node identification (80.5% vs. 61.0%) and was the sole detecting tracer in 39.0% of cases. Using methylene blue alone would have reduced the retrieval rate to 51.0% (*p* = 0.001). ICG identified the clipped node in all six patients with axillary micrometastases (ypN1mi). Clipped node non-retrieval occurred in eight patients, all of whom had negative SLNB findings and did not undergo completion axillary lymph node dissection. Recurrence rates were similar between the retrieval and non-retrieval groups (17.1% vs. 12.5%, *p* = 1.000), and no isolated axillary recurrences were observed. Baseline tumour size was the only independent predictor of disease-free survival in multivariable analysis (HR 2.05, 95% CI 1.20–3.51; *p* = 0.008). ***Conclusions***: Radiotracer-free axillary staging using ICG and methylene blue achieved a high clipped node retrieval rate in patients with ycN0 breast cancer after neoadjuvant chemotherapy. ICG significantly improved clipped node identification compared with methylene blue alone and identified the clipped node in all patients with residual nodal micrometastatic disease. Among patients with negative SLNB findings, clipped node non-retrieval was not associated with higher recurrence rates or worse disease-free survival during follow-up. However, given the small number of clipped node non-retrieval cases (n = 8) and recurrence events (n = 8), these findings should be interpreted with caution and require confirmation in larger prospective studies. These findings support the feasibility of a radiotracer-free dual-tracer approach in settings where radiotracer-based techniques are not available. Prospective multicentre studies are warranted to validate both the oncological performance of radiotracer-free axillary staging and the clinical implications of clipped node non-retrieval.

## Simple Summary

Neoadjuvant chemotherapy (NAC) can eliminate cancer from the axillary lymph nodes in many patients with initially node-positive breast cancer, allowing less extensive surgery. To ensure accurate staging after treatment, suspicious lymph nodes are marked with clips before NAC and should ideally be removed during surgery. Many centres rely on radiotracers or magnetic seeds for this purpose, but these technologies are not universally available. In this study, we evaluated a radiotracer-free approach using indocyanine green (ICG) and methylene blue to identify sentinel lymph nodes and retrieve the clipped node. We found that ICG significantly improved clipped node detection compared with methylene blue alone and identified all patients with residual nodal micrometastatic disease. Importantly, failure to retrieve the clipped node was not associated with higher recurrence rates during follow-up in patients with negative sentinel lymph node biopsy findings. These findings suggest that this dual-tracer technique is a practical and effective alternative for centres without access to radiotracer-based technologies, although larger prospective studies are needed to confirm these results.

## 1. Introduction

Neoadjuvant chemotherapy (NAC) frequently achieves axillary downstaging in patients with initially node-positive breast cancer, allowing for less extensive axillary surgery in selected patients who convert to clinically node-negative (ycN0) status [1,2]. Targeted axillary staging approaches incorporating retrieval of the previously biopsied metastatic lymph node have improved the accuracy of post-NAC axillary assessment and are increasingly used in routine practice [3,4,5,6,7]. Most targeted axillary staging techniques rely on radiotracer and magnetic localization technologies to facilitate identification of the clipped lymph node. However, access to these resources remains limited in many institutions worldwide because of logistical, regulatory, and economic constraints [8]. Dual-tracer mapping with indocyanine green (ICG) and methylene blue represents a practical radiotracer-free alternative. Although ICG has demonstrated superior sentinel lymph node detection compared with methylene blue alone [9,10], its contribution to clipped node retrieval in radiotracer-free axillary staging has not been well characterised. In addition, clipped node retrieval is not always successful despite technically adequate axillary staging [11,12,13]. The clinical significance of clipped node non-retrieval, particularly in patients with negative sentinel lymph nodes, remains unclear. Therefore, this study aimed to evaluate the performance of radiotracer-free axillary staging using ICG and methylene blue in patients with ycN0 breast cancer after NAC. We further assessed the contribution of each tracer to clipped node identification and explored the clinical outcomes associated with clipped node non-retrieval.

## 2. Materials and Methods

### 2.1. Study Design and Patient Selection

This retrospective single-centre cohort study was conducted at the University of Health Sciences Istanbul Bagcilar Training and Research Hospital. Consecutive patients who underwent surgery between January 2021 and December 2024 were considered eligible if they met the following criteria: (1) biopsy-confirmed cN1 invasive breast carcinoma; (2) pre-treatment ultrasound-guided clip placement in a biopsy-proven metastatic axillary lymph node; (3) completion of standard NAC (radiotherapy, when indicated, was administered in the adjuvant setting; no patient received neoadjuvant radiotherapy); (4) documented ycN0 status based on post-treatment axillary ultrasonography; (5) dual-tracer sentinel lymph node biopsy (SLNB) performed using ICG and methylene blue; (6) a minimum postoperative follow-up of 12 months. Patients with cN2–N3 disease, distant metastasis, inflammatory breast cancer, prior axillary surgery, or incomplete clinical data were excluded. The study protocol was approved by the Institutional Review Board of the University of Health Sciences Istanbul Bagcilar Training and Research Hospital (approval date: 25 September 2025; approval number: 12/05/082) and was conducted in accordance with the Declaration of Helsinki.

### 2.2. Surgical Technique and Tracer Documentation

ICG (Verdye®, Diagnostic Green GmbH, Aschheim, Germany; 1.25 mg/mL, total volume 5 mL) was administered via periareolar injection, and methylene blue (Methylene Blue 1%, Osel İlaç, İstanbul, Türkiye; 2 mL) was injected subareolarly approximately 10 min prior to skin incision. Axillary exploration was performed using a near-infrared fluorescence imaging system (SPY-PHI, Stryker, Kalamazoo, MI, USA) to enable real-time identification of fluorescent lymph nodes. All nodes demonstrating fluorescence, defined as a signal clearly distinguishable from background tissue, were excised, together with any blue-stained nodes. If the clipped node was not identified among the retrieved sentinel lymph nodes or by intraoperative palpation or direct visualisation, no additional axillary dissection was performed solely to locate or retrieve the clipped node. Successful retrieval of the clipped node was confirmed by intraoperative palpation and specimen radiography. For each case, the surgeon prospectively recorded whether the clipped node was identified by ICG alone, methylene blue alone, both tracers, or neither. All excised sentinel lymph nodes underwent intraoperative frozen-section evaluation followed by definitive histopathological assessment.

### 2.3. Outcome Definitions

Breast pathological complete response (pCR) was defined as Miller–Payne grade 5, corresponding to the absence of residual invasive tumour in the breast (ypT0). In this study, breast pCR required the complete absence of residual invasive carcinoma (ypT0); residual ductal carcinoma in situ (DCIS) was not classified as pCR. This definition is more stringent than the ypT0/is criterion used by several contemporary studies, which also permits residual in situ disease, and it was applied independently of the separately reported axillary (nodal) response. Axillary pathological response was classified as ypN0 (no residual tumour cells on final paraffin histology, including isolated tumour cells) or ypN1mi (micrometastatic disease, defined as tumour deposits >0.2 mm and ≤2 mm). A negative SLNB was defined as the absence of both macrometastatic and micrometastatic disease on intraoperative frozen section and definitive paraffin histopathological evaluation. Nodes containing only isolated tumour cells (ypN0[i+]) were classified as ypN0 and managed without completion axillary lymph node dissection (ALND), in accordance with institutional protocols. Disease-free survival (DFS) was defined as the interval from the date of initial histopathological diagnosis to the first occurrence of locoregional or distant recurrence, or death from any cause. Clipped node non-retrieval was defined as the failure to identify and excise the clipped lymph node despite technically adequate dual-tracer mapping and standard intraoperative assessment.

### 2.4. Statistical Analysis

Categorical variables were compared with Fisher’s exact or chi-square tests, as appropriate. Clipped node retrieval according to the tracer was evaluated using Fisher’s exact test at the cohort level and McNemar’s test within the retrieved subgroup. Continuous data were analysed with the Mann–Whitney U or independent-samples *t*-test. Disease-free survival was estimated by the Kaplan–Meier method and compared between groups using the log-rank test. Predictors of disease-free survival were assessed with univariable and multivariable Cox proportional hazards regression. Due to the small number of recurrence events (n = 8), multivariable logistic regression with L2 penalization was additionally performed for the binary recurrence outcome. All tests were two-sided and a *p*-value < 0.05 was considered statistically significant. Analyses were conducted using SPSS version 29 (IBM Corp., Armonk, NY, USA).

## 3. Results

### 3.1. Patient Characteristics

Forty-nine patients met the inclusion criteria. The median age was 53 years (range 27–79), mean BMI was 28.9 ± 5.2 kg/m^2^, and 36 patients (73.5%) were postmenopausal. Invasive ductal carcinoma was the predominant histology (89.8%). The most common molecular subtype was Luminal B HER2-negative (n = 30; 61.2%), and lymphovascular invasion was present in 27 patients (55.1%). Median follow-up was 41.6 months (25.5–51.3). Patient and tumour characteristics are presented in Table 1.

### 3.2. Pathological Response

Breast pathological complete response (pCR; Miller–Payne grade 5) was achieved in 14 patients (28.6%). pCR rates differed significantly according to molecular subtype (*p* = 0.0006), with the highest rates observed in HER2-positive tumours and no breast pCR observed in the small Luminal A subgroup. Lymphovascular invasion was associated with lower pCR rates (7.4% vs. 41.2%, *p* = 0.017). Axillary pathological assessment demonstrated ypN0 disease in 43 patients (87.8%) and residual micrometastatic disease (ypN1mi) in 6 patients (12.2%). No patient had residual macrometastatic nodal disease. Detailed pathological response data are presented in Table 2.

### 3.3. Dual-Tracer SLNB and Clipped Node Retrieval

A mean of 3.6 ± 0.9 sentinel lymph nodes (range, 2–6) were retrieved per patient. The clipped node was successfully identified in 41 of 49 patients (83.7%). Among these cases, ICG identified the clipped node in 33 patients (80.5%), whereas methylene blue identified it in 25 (61.0%). In 16 patients (39.0%), the clipped node was identified exclusively by ICG. At the cohort level, methylene blue alone would have resulted in a significantly lower clipped node retrieval rate (25/49; 51.0%) compared with dual-tracer mapping (41/49; 83.7%; Fisher’s exact *p* = 0.001). Within the subgroup of patients with successful retrieval, the difference between tracers was not statistically significant (McNemar *p* = 0.153). In the ypN1mi subgroup (n = 6), the clipped node was identified by ICG in all patients, whereas methylene blue alone failed to identify the clipped node in four cases. On multivariable logistic regression analysis, high Ki-67 (≥40%) showed a non-significant trend toward association with clipped node non-retrieval (OR 2.45, 95% CI 0.71–8.50; *p* = 0.157). Patients in the non-retrieval group had a higher mean number of ICG-positive lymph nodes than those in the retrieval group (3.6 vs. 2.8; *p* = 0.011). Detailed SLNB outcomes are presented in Table 3.

### 3.4. Clinical Outcomes According to Clipped Node Retrieval Status

All eight patients with clipped node non-retrieval had negative sentinel lymph nodes on both frozen section and final histology and did not undergo completion ALND. Recurrence occurred in 1 of these 8 patients (12.5%), compared with 7 of 41 (17.1%) in the retrieved group (Fisher’s exact *p* = 1.000; OR 0.69). No isolated axillary recurrences were observed in either group. Kaplan–Meier analysis showed no difference in disease-free survival between the non-retrieval and retrieved groups (log-rank *p* = 0.778). The four non-retrieval patients who achieved breast pCR remained event-free during follow-up (40.2–49.6 months).

### 3.5. Recurrence Patterns and Survival Outcomes

Eight recurrences (16.3%) were observed during follow-up: seven systemic and five locoregional (four patients had both). The anatomical sites of the locoregional recurrences and, where available, the anatomical level of the clipped node (Level I vs. Level II) are summarised in Supplementary Table S1. No recurrences occurred in the 14 patients who achieved breast pCR (*p* = 0.179 vs. non-pCR). Recurrence rates were similar between ypN0 and ypN1mi patients (16.3% vs. 16.7%; *p* = 1.000). An unexpectedly high recurrence rate was noted in the Luminal A subgroup (3/6; 50.0%) compared with all other subtypes (5/43; 11.6%; Fisher’s exact *p* = 0.047; OR 7.60). On multivariable logistic regression, baseline tumour size was the only independent predictor of recurrence (OR 2.41 per SD, 95% CI 1.12–5.22; *p* = 0.025). Recurrence data are presented in Table 4.

### 3.6. Survival Analysis

**Figure 1:**
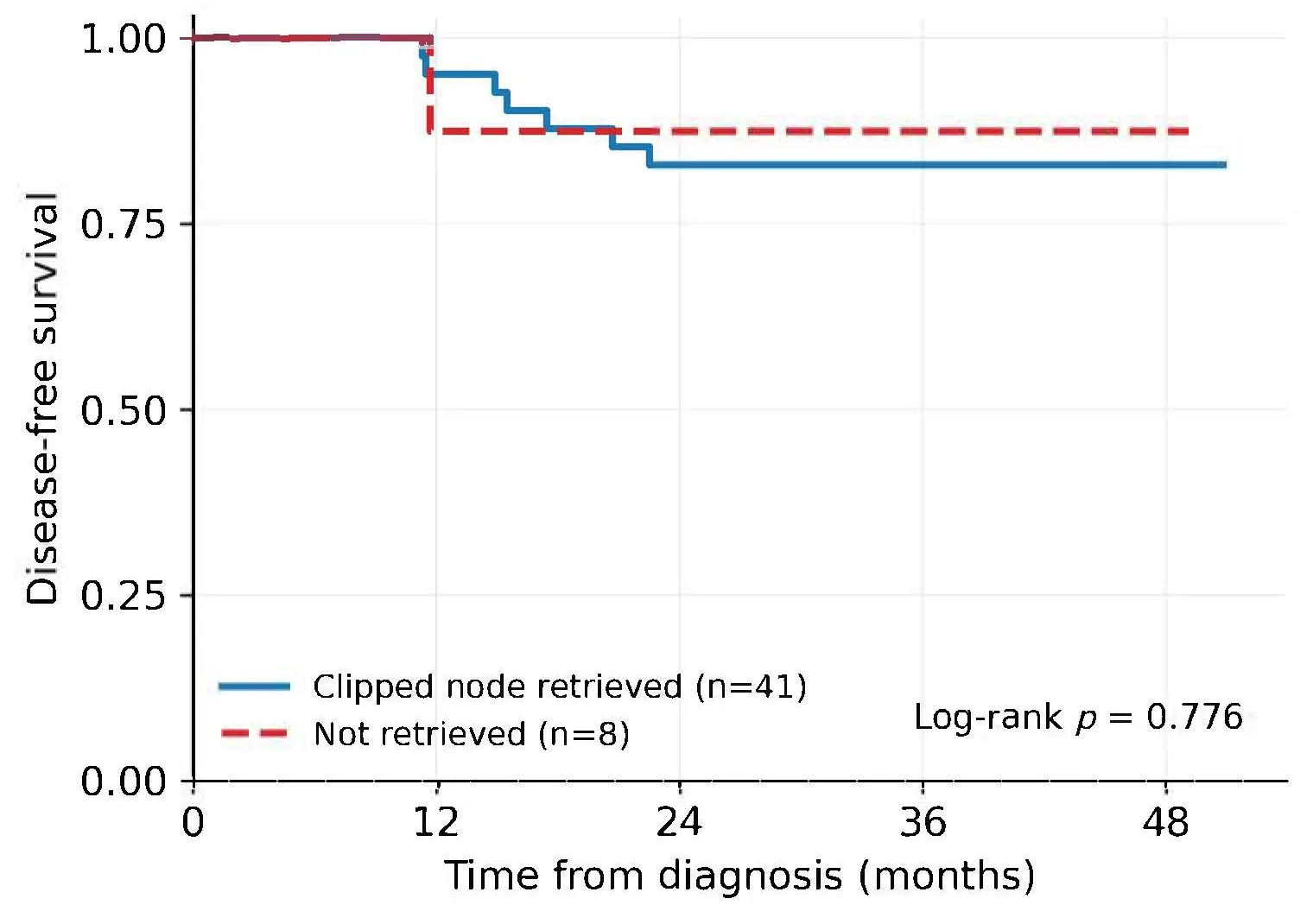
Kaplan–Meier disease-free survival according to clipped node retrieval versus non-retrieval. Kaplan–Meier curves comparing disease-free survival between patients with successful clipped node retrieval (n = 41) and those without clipped node retrieval (n = 8).

**Figure 2:**
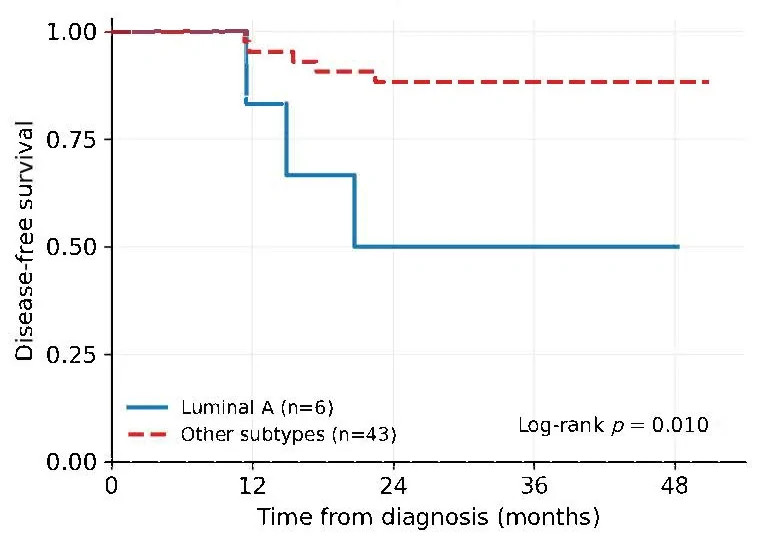
Kaplan–Meier disease-free survival according to Luminal A versus other molecular subtypes. Kaplan–Meier curves comparing disease-free survival between patients with Luminal A tumours and those with other molecular subtypes.

**Figure 3:**
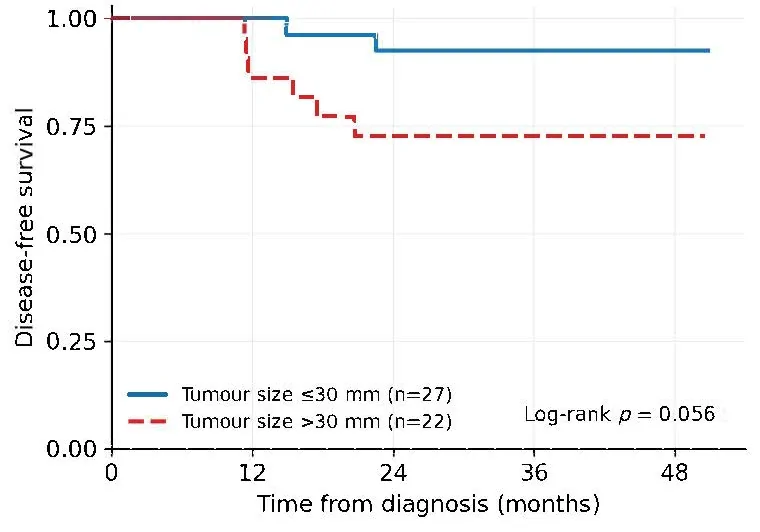
Kaplan–Meier disease-free survival according to tumour size (≤30 mm versus >30 mm). Kaplan–Meier curves comparing disease-free survival between patients with a baseline tumour size ≤30 mm and >30 mm.

Univariable and multivariable Cox proportional hazards regression analyses were performed to identify predictors of DFS. In univariable analysis, baseline tumour size demonstrated the strongest association with DFS (HR 3.00, 95% CI 1.55–5.80; *p* = 0.001). This association remained significant in the multivariable model after adjustment for clipped node retrieval status (HR 2.05, 95% CI 1.20–3.51; *p* = 0.008). Clipped node non-retrieval was not associated with DFS (HR 0.76, 95% CI not estimable; *p* = 0.727). All Cox regression analyses were considered exploratory due to the limited number of events (n = 8). Detailed results of the Cox regression analyses are provided in Supplementary Tables S2 (univariable analysis) and S3 (multivariable analysis). Kaplan–Meier curves illustrating DFS across key subgroups are shown in Figure 1, Figure 2 and Figure 3: Figure 1 shows clipped node retrieval versus non-retrieval; Figure 2 shows Luminal A versus other molecular subtypes; and Figure 3 shows tumour size ≤30 mm versus >30 mm. Kaplan–Meier curves for breast pCR and lymphovascular invasion (LVI) are provided as Supplementary Figures S1 and S2.

## 4. Discussion

This study provides clinically relevant evidence regarding the role of ICG in axillary staging without a radiotracer. In the absence of radiotracer availability, ICG demonstrated clear incremental value over methylene blue, with its omission associated with a substantial increase in clipped node non-retrieval rates (from 16.3% to 49.0%, *p* = 0.001). Moreover, when sentinel lymph nodes were negative following comprehensive dual-tracer assessment, failure to retrieve the clipped node was not associated with inferior oncological outcomes.

Although direct evidence specifically addressing ypN1mi patients after NAC remains limited, recent studies support the feasibility and diagnostic performance of ICG-based sentinel node mapping in the post-neoadjuvant setting [9,10,14]. In this context, our observation that ICG identified the clipped node in all six ypN1mi patients, whereas methylene blue alone failed in four of these six cases, suggests that the incremental value of ICG may be particularly relevant in patients with low-volume residual nodal disease. The superior performance of ICG in high-response cases may be related to treatment-induced changes in axillary lymphatic architecture. Following NAC, fibrosis and nodal regression may alter conventional lymphatic pathways, potentially reducing the reliability of blue dye mapping, while near-infrared fluorescence from ICG may remain detectable within reorganised tissue.

A non-significant trend toward higher clipped node non-retrieval rates was observed in patients with elevated Ki-67 levels (OR 2.45; *p* = 0.157) and the greater number of ICG-positive nodes in the non-retrieval group (*p* = 0.011) may further support a potential link between tumour response and altered lymphatic mapping patterns. One possible explanation is that tumours with a more pronounced response to NAC undergo greater nodal regression and fibrosis, which may alter lymphatic drainage pathways and contribute to technical challenges in clip localisation. However, these findings should be interpreted with caution given the limited sample size and lack of statistical significance. If validated in larger cohorts, pre-treatment tumour biology may represent a potential factor influencing the technical success of targeted axillary dissection.

Several prospective studies, including ACOSOG Z1071, SENTINA, and SN-FNAC, have evaluated the feasibility of SLNB in patients with initially node-positive breast cancer who convert to ycN0 status following neoadjuvant chemotherapy [1,15,16,17,18,19]. Collectively, these studies demonstrated that acceptable false-negative rates can be achieved when dual-tracer mapping is used, at least three sentinel lymph nodes are retrieved, and meticulous pathological assessment is performed. Similarly, the NSABP B-27 trial reported identification and false-negative rates comparable to those observed in patients undergoing upfront surgery, further supporting the feasibility of SLNB after NAC [20]. Concerns regarding false-negative rates subsequently led to the development of targeted axillary staging approaches incorporating retrieval of the previously biopsied metastatic lymph node. However, the clinical significance of clipped node non-retrieval remains unclear. Since failure to retrieve the clipped node effectively results in management by SLNB alone, understanding the oncological implications of clipped node non-retrieval remains clinically relevant. In this series, recurrence rates were similar between non-retrieval and retrieved groups (12.5% vs. 17.1%; *p* = 1.000), no isolated axillary recurrences occurred in either group, and Kaplan–Meier curves for disease-free survival were superimposable (log-rank *p* = 0.778). This is consistent with Boyle et al. [21], who reported no axillary nodal recurrences in either TAD- or SLNB-alone groups. Our clipped node retrieval rate of 83.7% is broadly comparable to that reported for radiotracer- and device-based targeted axillary staging. Series using iodine-125 radioactive seeds or radiocolloid have reported clipped/target node retrieval rates of approximately 90–98%, and magnetic seed (e.g., Magseed) localisation has achieved similarly high rates [2,4,5,6,22,23,24,25]. The somewhat lower rate in our radiotracer-free cohort is offset by avoiding radioactive and magnetic hardware, and the incremental value of ICG narrowed this gap, particularly in the micrometastatic subgroup [26]. A direct prospective comparison of radiotracer-free dual-tracer mapping against seed- and magnet-based techniques remains an important next step to position this approach within current practice.

A higher recurrence rate was observed in the Luminal A subgroup compared with the remaining molecular subtypes. However, this finding should be interpreted cautiously given the small number of Luminal A cases and recurrence events. Notably, all recurrences occurred in patients with suboptimal pathological response (Miller–Payne grade 2–3), supporting previous observations that response to neoadjuvant therapy is a major determinant of long-term outcome [27]. Beyond pathological response, the biological determinants of residual axillary disease after neoadjuvant chemotherapy warrant consideration. In a recent cohort of 262 patients, baseline node-positive status, hormone receptor-positive/HER2-negative (luminal) subtype, and larger post-treatment tumour size were independent predictors of high-burden residual nodal disease [25,28]. These findings are concordant with the elevated recurrence rate observed in our luminal subgroup and suggest that molecular subtype, rather than clip retrieval alone, may be an important driver of axillary and oncological outcome after NAC [29,30].

This study has several limitations. First, its retrospective single-centre design introduces the possibility of selection bias and unmeasured confounding. Second, ycN0 status was determined using institutional axillary ultrasonography, which may limit generalizability across centres. Moreover, the cohort was highly selected: eligibility required cN1 disease, pre-treatment clip placement in the biopsied node, ycN0 conversion documented on ultrasound, successful dual-tracer mapping, and at least 12 months of follow-up. Patients with cN2-3 disease, incomplete mapping, or shorter follow-up were not represented. These criteria enrich the cohort for favourable, treatment-responsive disease and limit the extent to which the findings can be generalised to unselected node-positive populations or to centres using different imaging and mapping protocols. Third, the small number of recurrence events (n = 8) and clipped node non-retrieval cases (n = 8) limits the statistical power of subgroup analyses and increases the risk of overfitting in multivariable models; therefore, all regression analyses should be considered exploratory and hypothesis-generating. In addition, the median follow-up of 41.6 months may underestimate late recurrences, particularly in hormone receptor-positive disease. Finally, interpretation of the oncological outcomes should occur within the context of contemporary multimodality treatment, including systemic therapy and adjuvant radiotherapy, both of which may have contributed to the low rates of axillary recurrence observed in this cohort. In particular, most patients (85.7%) received adjuvant radiotherapy, which may itself have sterilised residual axillary disease and thereby confounded the apparently low rate of axillary recurrence; the oncological safety of clipped node non-retrieval therefore cannot be attributed to the surgical approach alone. These findings should also be interpreted within the context of evolving axillary management: the NRG Oncology/NSABP B-51/RTOG 1304 trial showed that regional nodal irradiation did not reduce invasive recurrence in patients converting to ypN0 after neoadjuvant chemotherapy [16]. As regional nodal irradiation is de-escalated in selected responders, the relative contribution of accurate surgical staging, and thus of clipped node retrieval, may increase and warrants dedicated prospective evaluation. Prospective multicentre studies, particularly in settings where radioisotope tracers are unavailable, are needed to validate both the performance of radiotracer-free axillary staging and the clinical implications of clipped node non-retrieval. To address these limitations, several concrete steps are warranted: adequately powered prospective multicentre studies with pre-specified recurrence and survival endpoints; standardised and ideally centrally reviewed criteria for post-treatment ycN0 assessment to reduce inter-centre variability; extended follow-up of at least five years to capture the late recurrences characteristic of hormone receptor-positive disease; and, where feasible, enrolment within international registries such as AXSANA (EUBREAST-03) to accrue sufficient clipped node non-retrieval events for robust oncological analysis.

## 5. Conclusions

Radiotracer-free axillary staging using ICG and methylene blue achieved a high clipped node retrieval rate in patients with ycN0 breast cancer after neoadjuvant chemotherapy. ICG provided substantial incremental value beyond methylene blue alone and identified the clipped node in all patients with residual nodal micrometastatic disease. Among patients with negative SLNB findings, clipped node non-retrieval was not associated with higher recurrence rates or inferior disease-free survival during follow-up. Because these observations are based on a small number of events, they should be interpreted as preliminary and hypothesis-generating. These findings support the feasibility of a radiotracer-free dual-tracer approach in settings where radiotracer-based techniques are not available. Larger prospective multicentre studies are required to validate both the oncological performance of radiotracer-free axillary staging and the clinical implications of clipped node non-retrieval.

## Figures and Tables

**Table 1:** Patient and tumour characteristics (n = 49).

Characteristic	n (%) or Median (Range)
Dem ographics	
Age, years—median (range)	53 (27–79)
BMI, kg/m^2^—mean ± SD	28.9 ± 5.2
Premenopausal	13 (26.5)
Postmenopausal/perimenopausal	36 (73.5)
Pathology and staging	
Invasive ductal carcinoma	44 (89.8)
Lymphovascular invasion-positive	27 (55.1)
Baseline tumour size, mm—mean ± SD	33.8 ± 14.5
Baseline Ki-67, %—mean ± SD	41.4 ± 26.2
cT1/cT2/cT3/cT4	6/36/5/2
Molecular subtype	
Luminal A	6 (12.2)
Luminal B HER2-negative	30 (61.2)
HER2-enriched	5 (10.2)
Luminal B HER2-positive	5 (10.2)
Triple-negative	3 (6.1)
Treatment	
Breast-conserving surgery	29 (59.2)
Mastectomy	20 (40.8)
Adjuvant radiotherapy	42 (85.7)
Follow-up, months—median (range)	41.0 (25.0–50.8)

**Table 2:** Pathological response and recurrence outcomes.

	n (%)	Breast pCR	Recurrence	*p*
*Breast response (Miller–Payne grade)*				
Grade 1—no response	1 (2.0)	—	0/1	—
Grade 2—minimal	6 (12.2)	—	3/6 (50%)	—
Grade 3—partial	19 (38.8)	—	3/19 (16%)	—
Grade 4—marked	9 (18.4)	—	2/9 (22%)	—
Grade 5—pCR (ypT0)	14 (28.6)	14/14	0/14 (0%)	0.179 †
*Axillary pathological response*				
ypN0 (no micrometastases)	43 (87.8)	—	7/43 (16.3%)	—
ypN1mi (micrometastases)	6 (12.2)	—	1/6 (16.7%)	1.000 ‡
Macroscopic nodal disease (ypN1mac)	0 (0)	—	—	—
*Breast pCR by molecular subtype (chi-square p = 0.0006)*				
Luminal A	6 (12.2)	0/6 (0%)	3/6 (50.0%)	—
Luminal B HER2-negative	30 (61.2)	5/30 (16.7%)	4/30 (13.3%)	—
HER2-enriched	5 (10.2)	3/5 (60.0%)	1/5 (20.0%)	—
Luminal B HER2-positive	5 (10.2)	5/5 (100%)	0/5 (0%)	—
Triple-negative	3 (6.1)	1/3 (33.3%)	0/3 (0%)	—

† Fisher’s exact vs. non-pCR group. ‡ Fisher’s exact vs. ypN0 group. Luminal A.

**Table 3:** Dual-tracer SLNB outcomes, clipped node retrieval, and non-retrieval subgroup.

Variable	Clipped Node Retrieved (n = 41)	Clipped Node Not Retrieved (n = 8)
SLNB technical outcomes		
Total nodes retrieved, mean ± SD (range)	3.6 ± 0.9 (2–6)	4.1 ± 0.8
ICG-detected nodes, mean	2.8	3.6 *
Methylene blue-detected nodes, mean	1.9	1.8
Clip detection by tracer (retrieved group, n = 41)		
ICG-detected clipped node	33/41 (80.5%)	n/a
Methylene blue-detected clipped node	25/41 (61.0%)	n/a
Exclusively ICG-detected clipped node	16/41 (39.0%)	n/a
Exclusively methylene blue-detected	8/41 (19.5%)	n/a
McNemar test *p* (ICG vs. MB, within retrieved group)	*p* = 0.153	—
ICG+MB vs. MB-alone retrieval rate †	*41/49 (83.7%) vs. 25/49 (51.0%)*	Fisher’s *p* = 0.001; OR = 4.92
ICG clip detection in ypN1mi subgroup	6/6 (100%) ‡	n/a
Non-retrieval subgroup (n = 8)		
Breast pCR (Miller–Payne Grade 5)	10/41 (24.4%)	4/8 (50.0%)
Partial/minimal breast response	31/41 (75.6%)	4/8 (50.0%)
Axillary status (ypN0)	41/41 (100%)	8/8 (100%)
Micrometastases (ypN1mi)	6/41 (14.6%)	0/8 (0%)
Completion ALND performed	0/41 (0%)	0/8 (0%)
Any recurrence	7/41 (17.1%)	1/8 (12.5%)
Isolated axillary recurrence	0	0
Fisher’s exact *p* (recurrence)	Reference	*p* = 1.000; OR = 0.69
Log-rank *p* (DFS)	Reference	*p* = 0.778
Follow-up, months (range)	39.1 (25.0–50.8)	46.5 (39.7–50.4)
Non-retrieval predictors (multivariable logistic regression) §		
Ki-67 ≥ 40%—OR 2.45 (95% CI 0.71–8.50)	*p* = 0.157	Trend

* *p* = 0.011 vs. retrieved group (Mann–Whitney). † Evaluated across all 49 patients. ‡ Methylene blue alone would have identified 2/6 clipped nodes in the micrometastasis subgroup. § EPV = 4; exploratory.

**Table 4:** Recurrence analysis and multivariable logistic regression.

Comparison	Group 1	Group 2	*p*	OR (95% CI)
Recurrence by breast pathological response				
Breast pCR (MP5) vs. non-pCR	0/14 (0%)	8/35 (22.9%)	0.179	—
Miller–Payne ≥4 vs. ≤3	2/23 (8.7%)	6/26 (23.1%)	0.153	0.32 (0.06–1.77)
Recurrence by axillary pathological response				
ypN0 vs. ypN1mi	7/43 (16.3%)	1/6 (16.7%)	1.000	0.97 (0.10–9.41)
Recurrence by clipped node retrieval				
Retrieved vs. not retrieved	7/41 (17.1%)	1/8 (12.5%)	1.000	1.44 (0.16–13.1)
Log-rank *p* (DFS)	—	—	0.778	—
Recurrence by molecular subtype				
Luminal A vs. all others	3/6 (50.0%)	5/43 (11.6%)	0.047	7.60 (1.21–47.8)
Luminal B HER2-neg vs. others	4/30 (13.3%)	4/19 (21.1%)	0.470	0.58 (0.13–2.59)
LVI-positive vs. -negative	7/27 (25.9%)	1/17 (5.9%)	0.125	5.60 (0.62–50.4)
Multivariable logistic regression—predictors of recurrence (n = 49, events = 8) *				
*Baseline tumour size (per SD)*	*OR = 2.41*	*95% CI 1.12–5.22*	*0.025*	*Independent*
Luminal A subtype	OR = 2.36	95% CI 0.54–10.37	0.257	n.s.
LVI-positive	OR = 1.58	95% CI 0.36–6.87	0.545	n.s.
Miller–Payne grade (continuous)	OR = 0.57	95% CI 0.23–1.44	0.232	n.s.

* L2-penalised logistic regression; variables entered with univariate *p* < 0.20; EPV = 2.0 per variable (hypothesis-generating). OR = odds ratio; CI = confidence interval; DFS = disease-free survival; n.s. = not significant.

## Data Availability

The data presented in this study are not publicly available because they contain information that could compromise the privacy of research participants. Data are available from the corresponding author upon reasonable request and subject to institutional approval.

## Supplementary Materials

The following supporting information can be downloaded at: https://www.mdpi.com/article/10.3390/cancers18162550/s1, Supplementary Table S1. Recurrence patterns and clipped node anatomical level in patients with disease recurrence (n = 8); Table S2. Univariate Cox regression—disease-free survival; Table S3. Kaplan–Meier estimates of disease-free survival according to clinicopathological subgroups; Figure S1. Disease-free survival according to breast pathological complete response (pCR). Figure S2. Disease-free survival according to lymphovascular invasion (LVI) status.
