# Supplementary material for: Radiotracer-Free Axillary Staging with ICG and Methylene Blue After Neoadjuvant Chemotherapy: Does Failure to Retrieve the Clipped Node Matter?
Source: Cancers (Basel). 2026 Aug 8;18(16):2550. doi: 10.3390/cancers18162550 (PMC13511066; doi:10.3390/cancers18162550)
Supplement: Supplementary file 1 [file cancers-18-02550-s001.zip › cancers-4432888-supplementary.pdf]

Supplementary Materials

Table S1. Recurrence patterns and clipped node anatomical level in patients with disease recurrence (n = 8).

Case	C1	C2	C3	C4	C5	C6	C7	C8
Age (years)	50	58	63	27	50	66	65	45
Molecular subtype	Luminal A	Luminal A	Luminal B HER2−	Luminal B HER2−	Luminal B HER2−	Luminal B HER2−	Luminal B HER2−	Triple-negative
Miller–Payne	3	3	2	1	2	3	2	3
Breast pCR	No	No	No	No	No	No	No	No
LVI	Yes	Yes	Yes	Yes	Yes	Yes	Yes	Yes
Recurrence pattern	Systemic	Systemic	Systemic	Loco+syst	Locoregional	Systemic	Loco+syst	Loco+syst
Time to locoregional (mo)	–	–	–	–	15.5	–	11	22.5
Time to systemic (mo)	15	20.5	17.5	11.5	–	15	22.5	22.5
Site of recurrence [a]	Bone	Bone	Bone+Liver	Bone+Liver	Ipsilateral axillary LN	Liver	Chest wall+Liver	Chest wall+Bone
Clipped node level [a]	I	I	II	I	I	II	I	I
Isolated axillary recurrence	No	No	No	No	No	No	No	No
Death	No	No	No	No	Yes	No	No	No

[a] Site of recurrence and clipped node level were obtained from the source clinical records. For patients with locoregional recurrence the anatomical site is reported; for systemic-only recurrence the first site of distant metastasis is shown. Level refers to the anatomical level (I or II) of the retrieved clipped node. pCR, pathological complete response; LVI, lymphovascular invasion; Loco+syst, locoregional plus systemic.

Table S2. Univariate Cox regression — Disease-Free Survival.

Variable	HR	95% CI	Cox p	Log-rank
**Pathological response**				
Miller-Payne grade (continuous, 1–5)	0.532	0.153–1.849	0.321	0.192
Breast pCR (Grade 5) — see note*	—	—	—	0.059
**Axillary findings**				
Clipped node non-retrieval	0.780	0.089–6.858	0.823	0.778
Micrometastasis (ypN1mi)	2.953	0.267–32.657	0.377	0.993
**Tumor biology**				
LVI positive	1.282	0.146–11.269	0.823	0.050 †
Luminal A subtype	1.080	0.239–4.889	0.920	0.011 **
Baseline tumor size (per SD)	0.553	0.286–1.067	0.077 †	0.056 †

Ki-67 (per SD)	0.990	0.545–1.797	0.973	0.260
cT3–4 (>5 cm) vs cT1–2 (≤5 cm) — see note†	—	—	—	0.075 †

* Breast pCR (Miller-Payne Grade 5) could not be entered into Cox regression due to complete separation (zero events in the pCR group; n = 14); log-rank p reported. † cT stage could not be modeled by Cox regression due to sparse data (cT1 subgroup: 0 events); Fisher exact OR = 5.55, p = 0.075. HR = hazard ratio; CI = confidence interval; LVI = lymphovascular invasion; SD = standard deviation. † p < 0.10; ** p < 0.05. All results exploratory (EPV = 8, events-per-variable ratio < 10).

Table S3. Kaplan–Meier estimates of disease-free survival according to clinicopathological subgroups.

Group	n	DFS at 12 mo	DFS at 24 mo	Log-rank p
**Clipped node retrieval**				
Retrieved	41	95.1%	87.8%	0.778
Not retrieved	8	87.5%	87.5%	—
**Pathological response**				
Breast pCR (Miller-Payne Grade 5)	14	100%	100%	0.059 †
Non-pCR (Grade 1–4)	35	91.4%	77.1%	—
**Tumor size**				
≤30 mm (below median)	27	96.3%	92.6%	0.056 †
>30 mm (above median)	22	90.9%	72.7%	—
**Molecular subtype**				
Luminal A	6	83.3%	50.0%	0.011 **
All other subtypes	43	97.7%	93.0%	—
**Lymphovascular invasion**				
LVI positive	27	96.3%	74.1%	0.107
LVI negative	17	94.1%	94.1%	—

DFS = disease-free survival, calculated from the date of initial histopathological diagnosis to any recurrence event (local or systemic) or last follow-up. Median follow-up: 41.0 months (range 25.0–50.8). † p < 0.10; ** p < 0.05.

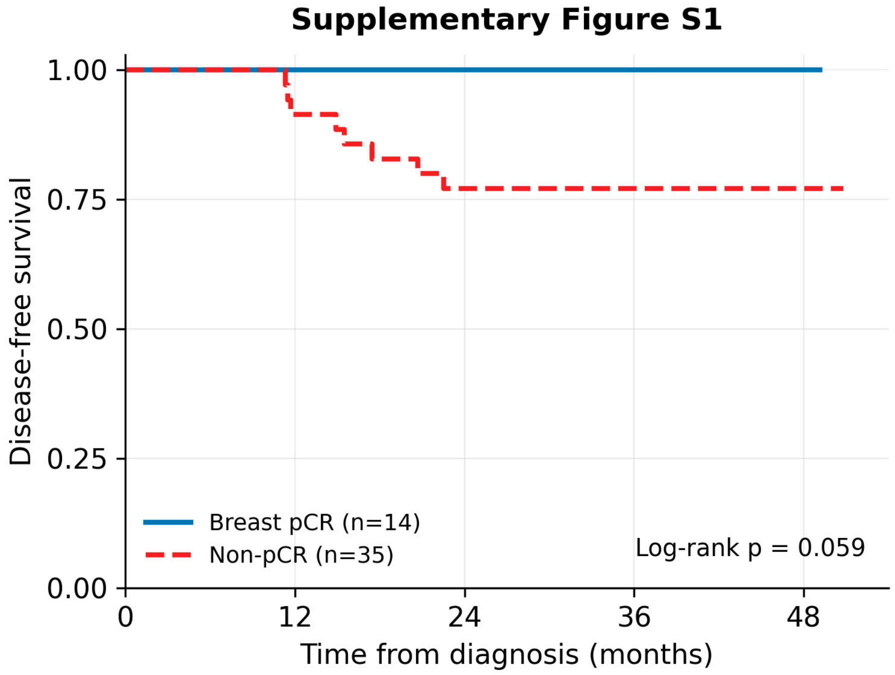

Figure S1. Kaplan–Meier analysis of disease-free survival according to breast pathological complete response (pCR). Kaplan–Meier curves showing disease-free survival according to breast pathological complete response (pCR). Patients achieving breast pCR (Miller–Payne grade 5; n = 14) remained free of recurrence during follow-up, whereas recurrence events occurred only in the non-pCR group (n = 35). The difference did not reach statistical significance (log-rank p = 0.059).

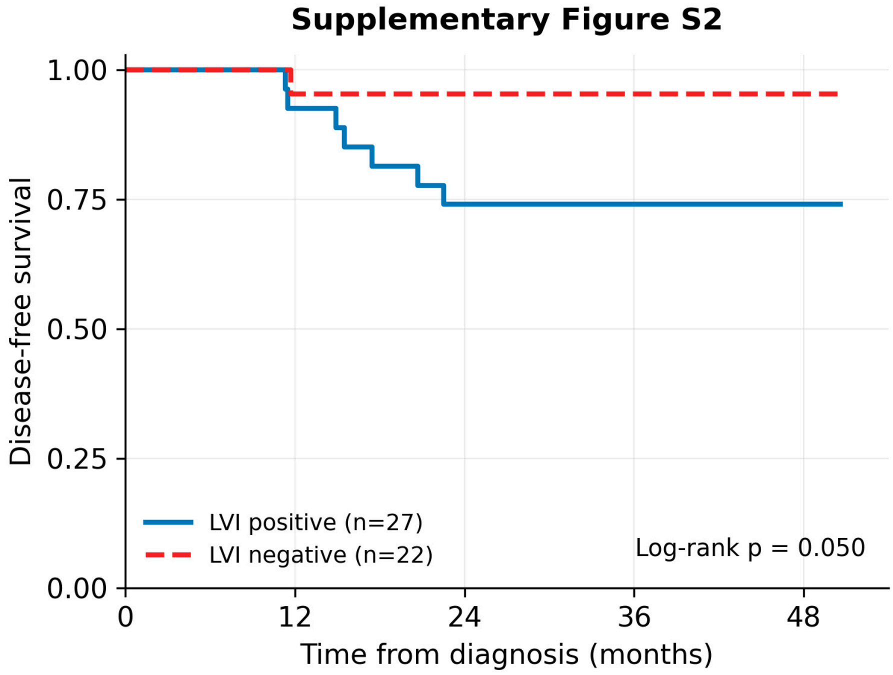

Figure S2. Kaplan–Meier analysis of disease-free survival according to lymphovascular invasion (LVI) status. Kaplan–Meier curves showing disease-free survival according to lymphovascular invasion (LVI) status. Disease-free survival was compared between patients with LVI-positive (n = 27) and LVI-negative (n = 22) tumours (log-rank p = 0.050).
